# Obesity and Skull Base Manifestations: Clinical Implications and Management

**DOI:** 10.3390/jcm15176688

**Published:** 2026-08-28

**Authors:** Mohamad R. Chaaban, Jessica W. Grayson, Kristen O. Riley, Bradford A. Woodworth

**Affiliations:** 1 Department of Otolaryngology–Head and Neck Surgery, The Cleveland Clinic, Cleveland, OH 44195, USA; chaabam@ccf.org; 2 Department of Otolaryngology–Head and Neck Surgery, University of Alabama at Birmingham, Birmingham, AL 35294, USA; jgrayson@uabmc.edu; 3 Department of Neurosurgery, University of Alabama at Birmingham, Birmingham, AL 35294, USA; koriley@uabmc.edu

**Keywords:** obesity, skull base, spontaneous cerebrospinal fluid leak, cerebrospinal fluid rhinorrhea, idiopathic intracranial hypertension, intracranial pressure, encephalocele, obstructive sleep apnea, endoscopic skull base surgery, acetazolamide, lumbar drain, venous sinus stenting, skull base reconstruction

## Abstract

Obesity has become an important modifier of skull base disease. Although obesity is discussed in the context of obstructive sleep apnea, anesthesia risk, and wound healing, its most important skull base manifestation is often mediated through chronically elevated intracranial pressure. Spontaneous cerebrospinal fluid (CSF) leaks and skull base encephaloceles are now widely recognized as acquired defects that commonly arise in overweight or obese patients with idiopathic intracranial hypertension (IIH). This relationship has direct implications for diagnosis, surgical planning, reconstruction, postoperative management, and long-term recurrence prevention. Importantly, patients with active spontaneous CSF leaks may not display the classic signs of IIH because the leak itself may provide partial CSF diversion. Thus, the absence of papilledema or visual symptoms should not falsely reassure the clinician that intracranial pressure is normal. Obstructive sleep apnea may further contribute to intermittent intracranial pressure elevation through hypoxia, hypercapnia, and impaired venous drainage, and it creates unique perioperative challenges after endoscopic skull base reconstruction because positive airway pressure must be balanced against the need to protect the repair. This review summarizes the pathophysiology, clinical presentation, diagnostic evaluation, and management of obesity-associated skull base manifestations, with emphasis on spontaneous CSF leaks, encephaloceles, IIH, obstructive sleep apnea, endoscopic repair, intracranial pressure management, and postoperative airway considerations.

## 1. Introduction

Obesity is a systemic disease with consequences that extend well beyond cardiometabolic risk. In rhinology and skull base surgery, obesity modifies disease through several overlapping pathways: altered respiratory physiology, obstructive sleep apnea (OSA), impaired venous return, chronically elevated intracranial pressure (ICP), perioperative respiratory risk, and the mechanical effects of positive airway pressure therapy [1,2,3,4]. These pathways are particularly important in spontaneous cerebrospinal fluid (CSF) leak patients, where obesity is not simply a background demographic feature but a central element of disease pathophysiology [5,6,7,8,9].

Spontaneous CSF leaks were historically described as “idiopathic” because they developed without preceding trauma, tumor, or surgery. This terminology is increasingly inaccurate. A large body of literature now supports the concept that many spontaneous leaks are acquired pressure-related defects caused by idiopathic intracranial hypertension (IIH) [6,10,11,12]. The typical patient is an overweight or obese, middle-aged woman with radiographic findings of chronic intracranial hypertension, including empty sella, optic nerve sheath dilation, globe flattening, Meckel’s cave enlargement, arachnoid pits, skull base thinning, encephaloceles, and frequently more than one skull base defect [9,10,11,13,14,15]. This phenotype differs from classic IIH patients seen by neurologists and neuro-ophthalmologists, who often present earlier with headache, papilledema, and visual symptoms [1,2]. In the spontaneous CSF leak population, the underlying elevated ICP or IIH may be clinically occult before repair because the active leak can function as a partial pressure-diversion route. After successful closure, the pressure disorder may become more clinically apparent or more readily detectable [13,15,16].

The clinical implication is straightforward but important: spontaneous CSF leak repair should not be treated as a simple patch of a hole in the skull base. Durable success requires repair of the defect and management of the underlying pressure disorder that created it [7,17,18]. Obesity and OSA are central to that process. They influence who develops the defect, how the patient presents, how the defect should be reconstructed, how ICP should be monitored, how recurrence risk should be reduced, and how positive airway pressure therapy should be managed after surgery [3,4,19,20].

This review focuses on obesity-associated skull base manifestations, with particular emphasis on spontaneous CSF leaks, skull base encephaloceles, IIH, and OSA. The goal is to provide a practical framework for clinical evaluation and management while highlighting areas where additional prospective data are still needed.

## 2. Methods

This narrative review was developed from a targeted literature search of PubMed/MEDLINE and Google Scholar, supplemented by review of reference lists from relevant articles. Search terms included combinations of ‘spontaneous CSF leak,’ ‘cerebrospinal fluid rhinorrhea,’ ‘encephalocele,’ ‘idiopathic intracranial hypertension,’ ‘intracranial pressure,’ ‘obesity,’ ‘body mass index,’ ‘obstructive sleep apnea,’ ‘positive airway pressure,’ ‘venous sinus stenosis,’ ‘venous sinus stenting,’ and ‘endoscopic skull base repair.’ Priority was given to peer-reviewed clinical studies, systematic reviews, meta-analyses, large retrospective series, prospective observational studies, and clinically relevant skull base surgery literature. Articles were included when they addressed obesity, IIH, ICP, OSA, spontaneous skull base defects, endoscopic reconstruction, postoperative PAP management, or recurrence prevention. Articles were excluded if they focused exclusively on traumatic, neoplastic, or iatrogenic CSF leaks without relevance to obesity-associated or pressure-mediated disease. Because this was not designed as a systematic review, formal risk-of-bias scoring and pooled analysis were not performed.

Some of the pressure-monitoring data and management observations discussed in this review derive from prior publications from our own institution. Although these studies contribute clinically relevant information, they also reflect single-institution experience and may be influenced by referral patterns, surgical technique, patient selection, and institutional postoperative protocols. These findings should therefore be interpreted alongside the broader literature, and multicenter prospective studies are needed to validate generalizability.

All figures in this manuscript are derived from original clinical material obtained at the authors’ institutions. Patient images have been de-identified in accordance with institutional protocols.

## 3. Obesity, IIH, and Skull Base Remodeling

IIH is defined by elevated ICP without hydrocephalus, mass lesion, abnormal CSF composition, or another identifiable cause [1,2]. The disorder is strongly associated with obesity and classically affects women of childbearing age, although spontaneous CSF leak patients often present later in life [7]. The pathogenesis of IIH remains incompletely understood, but obesity likely contributes through several mechanisms, including increased intra-abdominal and intrathoracic pressure, impaired cerebral venous return, venous sinus stenosis or venous hypertension, altered CSF absorption, adipose-related inflammatory signaling, and hormonal/metabolic effects that may influence CSF dynamics [1,2,21].

The evidence linking obesity with spontaneous CSF leaks and IIH is derived from several complementary sources. Epidemiologic and retrospective cohort studies consistently demonstrate that spontaneous CSF leak patients are frequently overweight or obese and that this phenotype overlaps with IIH [5,7,8,10,22,23,24]. Radiographic studies further support a pressure-mediated mechanism by demonstrating empty sella, optic nerve sheath dilation, skull base thinning, arachnoid pits, encephaloceles, dilated Meckel’s Cave, venous sinus stenosis, and multiple defects in many affected patients [5,11,14,15]. Pressure-monitoring studies have shown that ICP may be elevated or may become more apparent after leak closure, supporting the concept that an active leak can partially decompress the intracranial compartment [6,10,12,25,26,27]. However, many studies are retrospective, and BMI is often confounded by sex, age, OSA, venous sinus stenosis, and other comorbidities. Therefore, obesity should be understood as a major clinical risk marker and disease modifier rather than as a solitary determinant of elevated ICP in every patient.

**Figure 1:**
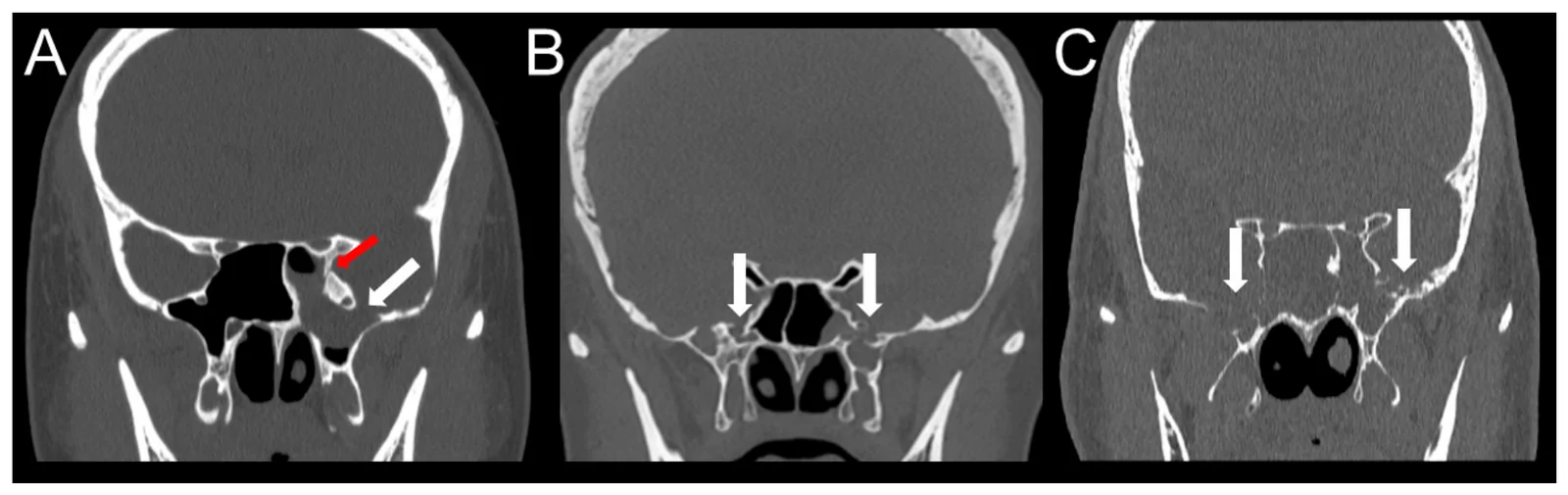
Sphenoid lateral recess defects with encephaloceles (white arrows) with several different presentations. The location of Sternberg’s canal is noted with the red arrow (**A**). Arachnoid pits from herniation into the bone are evident (**B**,**C**) with severe bone attenuation from high pressure.

The skull base is uniquely vulnerable to chronic pulsatile pressure. Over time, elevated ICP can remodel thin areas of bone and dura, creating arachnoid pits, thinning of the anterior skull base or lateral sphenoid recess, meningoencephaloceles, and ultimately CSF fistulas [28,29]. These lesions are not random. They occur where a thin skull base, aerated sinus cavity, pneumatized lateral recess, or preexisting area of relative weakness allows pressure to follow the pathway of least resistance [11,23,28,29,30]. This concept is especially important for lateral sphenoid recess defects. Although these lesions were historically attributed to a persistent Sternberg canal, objective radiographic and pressure data support an acquired mechanism from intracranial hypertension rather than a congenital canal (Figure 1) [11,28,31]. A true Sternberg canal would be medial to the superior orbital fissure and maxillary nerve, whereas most lateral recess sphenoid encephaloceles occur lateral to V2 and are accompanied by radiographic signs of chronically elevated ICP [13,31].

The association between obesity, IIH, and spontaneous CSF leaks is therefore not simply epidemiologic—it is biologic. Obesity increases the likelihood of sustained or intermittent intracranial hypertension; intracranial hypertension produces skull base remodeling; and skull base remodeling leads to encephalocele formation and CSF leak [6,7,8]. Once the leak forms, it can partially decompress the intracranial compartment, masking the classic neuro-ophthalmologic findings of IIH [13]. This is one reason these patients often present to otolaryngologists rather than neurologists.

## 4. Obstructive Sleep Apnea as a Modifier of Skull Base Disease

OSA is common in patients with obesity and has several plausible links to skull base pathology. During obstructive events, hypoxia and hypercapnia may cause cerebral vasodilation. Negative intrathoracic pressure swings and impaired venous return may further increase cerebral venous pressure. These physiologic events can produce transient ICP elevations during sleep [3,20,32]. Repetitive nocturnal ICP spikes, superimposed on a baseline predisposition to IIH, may contribute to skull base remodeling in susceptible patients.

The clinical literature supports an association between OSA and spontaneous CSF leaks, although the relationship is confounded by obesity, age, sex, and hypertension. A systematic review and meta-analysis found that patients with spontaneous CSF leaks had increased odds of having OSA compared with control cohorts [3]. However, most available studies were retrospective, and many relied on documented OSA diagnoses rather than systematic polysomnography. Thus, the true prevalence of OSA in spontaneous CSF leak patients is likely underestimated. Whether OSA independently causes spontaneous CSF leaks or acts through obesity and intracranial hypertension remains unresolved.

OSA also affects skull base surgery independent of leak pathogenesis. Patients with OSA are at increased risk for postoperative oxygen desaturation, respiratory events, and airway obstruction [4,19,33,34]. In endoscopic skull base surgery, nasal packing, splints, crusting, edema, and avoidance of postoperative positive airway pressure may worsen sleep-disordered breathing [4,19]. Conversely, early continuous positive airway pressure (CPAP) or bilevel positive airway pressure (BiPAP) may theoretically transmit pressure to the sinonasal cavity and skull base reconstruction, increasing concern for graft displacement, pneumocephalus, epistaxis, or postoperative CSF leak [33]. The literature remains limited and somewhat conflicting, but the practical issue is clear: OSA should be identified before surgery, and the timing of postoperative positive airway pressure should be individualized based on the severity of OSA, the nature of the reconstruction, the size and flow of the defect, and the patient’s respiratory risk.

For spontaneous CSF leak patients, the issue is even more complex. OSA may be part of the disease mechanism, yet CPAP may transiently increase CSF pressure and deliver positive pressure across the repair [19]. At the same time, withholding CPAP may worsen hypoxemia, hypercapnia, and nocturnal ICP spikes [4]. The goal should not be a rigid universal prohibition of CPAP; rather, the goal is coordinated perioperative management with otolaryngology, neurosurgery, anesthesia, and sleep medicine.

## 5. Clinical Phenotype and Presentation

**Figure 2:**
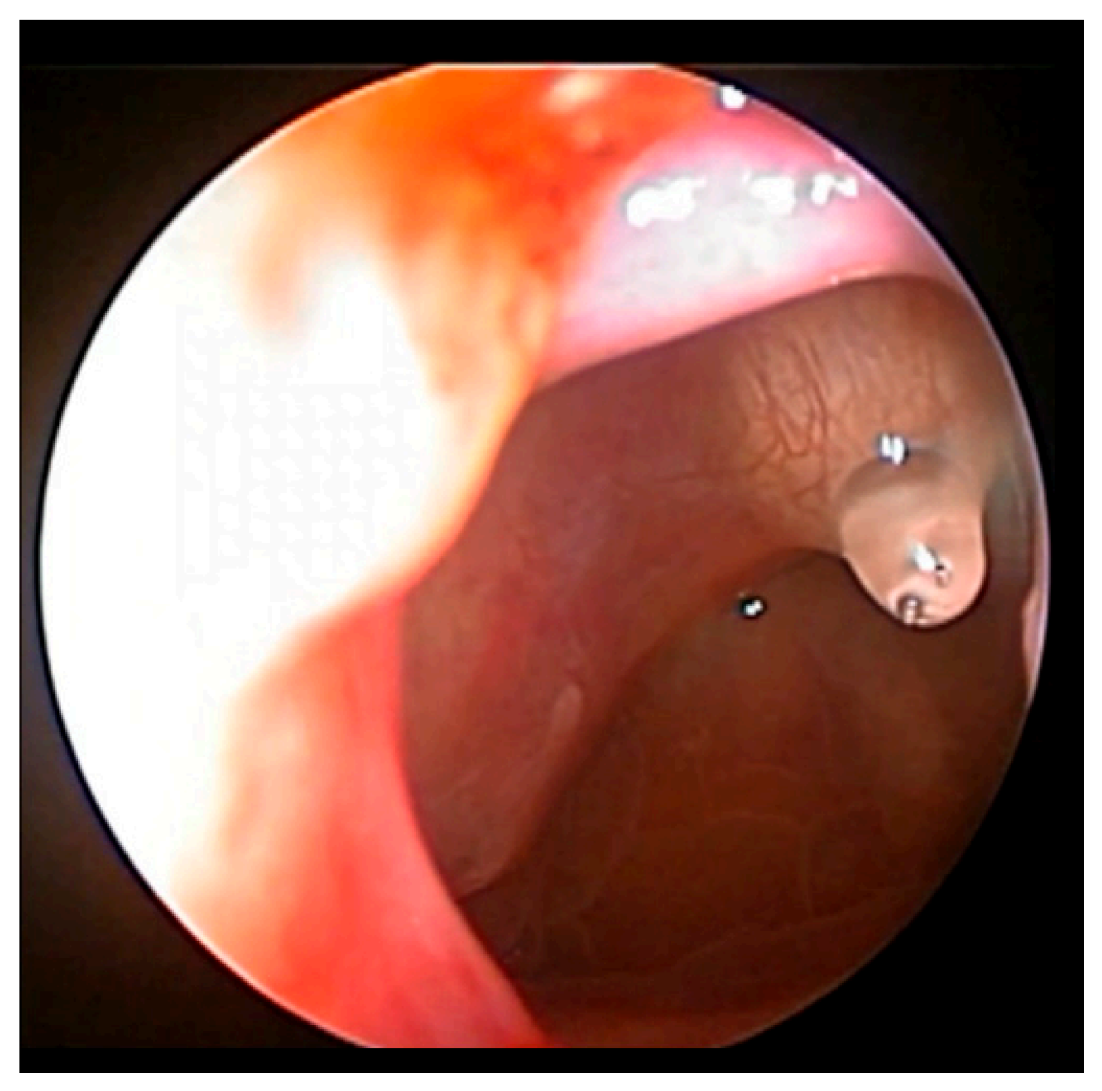
Transnasal endoscopic view of an active spontaneous CSF leak from the right planum sphenoidale.

The classic spontaneous CSF leak phenotype is an overweight or obese, middle-aged woman with unilateral watery rhinorrhea, often worse with bending forward, straining, or changes in head position (Figure 2) [5,10]. Patients may describe clear drainage, salty or metallic taste, cough from posterior drainage, recurrent “allergic rhinitis” that fails therapy, or repeated episodes of meningitis [22,24,35]. Some present only after imaging reveals an encephalocele or skull base defect. Others present with complications such as meningitis, pneumocephalus, or intracranial abscess [18].

The absence of visual symptoms or papilledema should not exclude IIH. In fact, this is one of the distinguishing features of the spontaneous CSF leak population. In our prior work, spontaneous CSF leak patients had elevated ICP but no preoperative papilledema. After the leak was repaired and the drain was clamped, ICP increased to levels comparable to IIH patients with papilledema [13]. This supports the concept that an active CSF leak functions as a partial pressure-diversion route. Once the route is closed, the underlying pressure disorder becomes unmasked.

**Figure 3:**
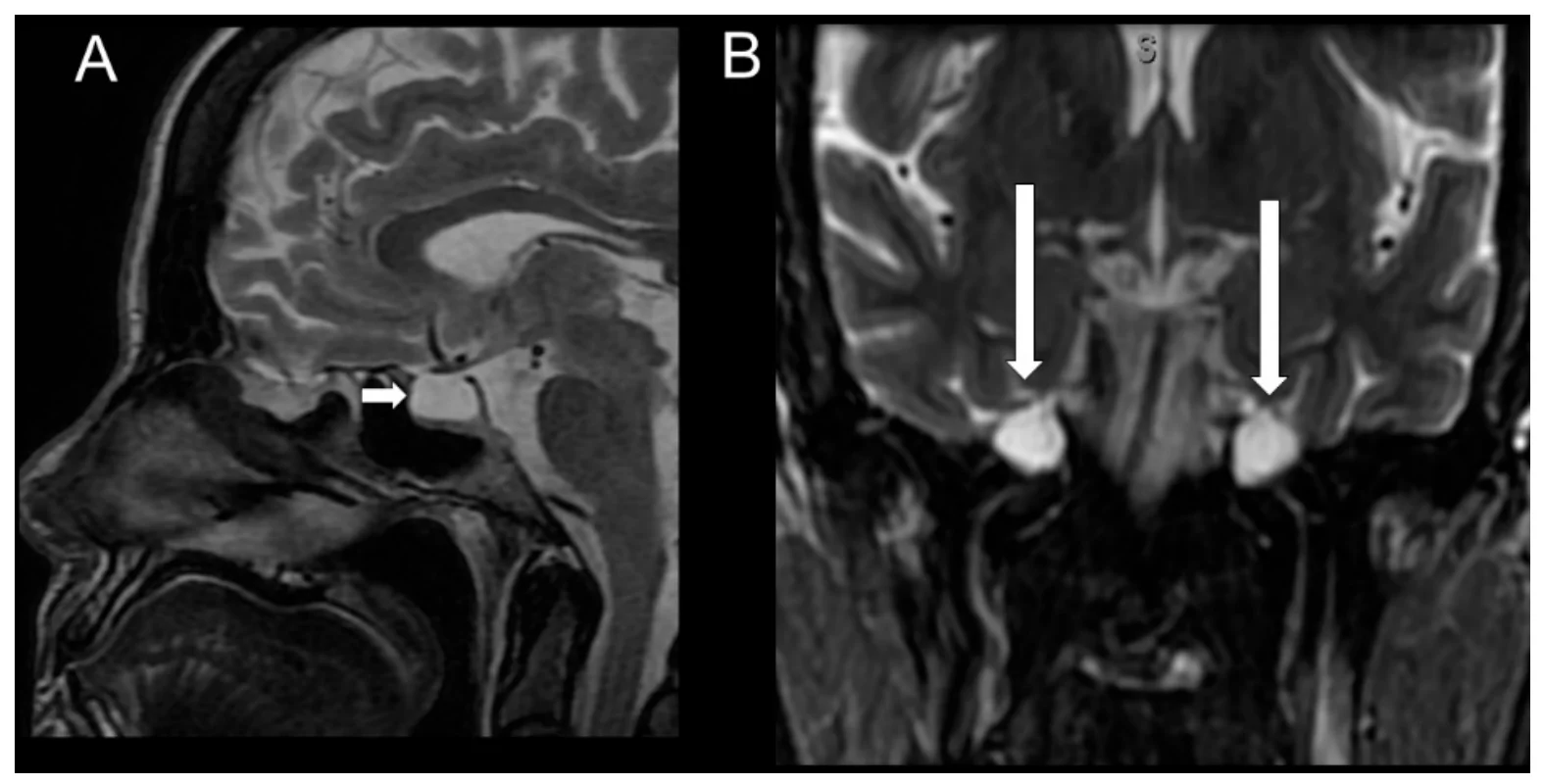
T2 weighted sagittal (**A**) and coronal (**B**) MRI scans demonstrating an empty sella (short arrow) and dilated Meckel’s caves (long arrows).

Radiographic findings are often the first clue. Empty sella, skull base thinning, multiple arachnoid pits, encephaloceles, dilated optic nerve sheaths, enlarged Meckel’s cave, venous sinus stenosis, and multiple defects should raise immediate concern for IIH (Figure 3) [7,9,11,13,14,21]. These findings are particularly important in patients with presumed traumatic or iatrogenic leaks who have failed prior repairs. If an obese patient has a recurrent leak after what should have been an adequate reconstruction, occult intracranial hypertension must be considered, regardless of the original etiology assigned to the defect [7,8,10].

Skull base manifestations may occur at several sites:

1. Anterior cranial fossa: cribriform, fovea ethmoidalis, planum sphenoidale, posterior table of the frontal sinus, or frontal recess. These patients most often present with CSF rhinorrhea [6,12,24,30,36,37,38].
2. Lateral sphenoid recess: often associated with extensive sphenoid pneumatization, arachnoid pits, and lateral encephaloceles. These defects frequently require expanded endoscopic access, including a transpterygoid approach [11,28,31].
3. Temporal bone/lateral skull base: spontaneous CSF otorrhea, middle ear effusion, conductive hearing loss, recurrent meningitis, or tegmen defects [32].
4. Multiple sites: multifocal defects should be considered evidence of a pressure disorder until proven otherwise [6,10,12,24].

## 6. Diagnostic Evaluation and Preoperative Assessment (Table 1)

Evaluation begins with suspicion. Clear unilateral rhinorrhea in an obese patient, particularly a middle-aged woman, should prompt consideration of CSF leak even when the history suggests rhinitis [35]. The same is true for recurrent meningitis, unexplained middle ear effusion, or radiographic encephalocele [18,32].

### 6.1. Laboratory Confirmation

When fluid is available, beta-2 transferrin remains a highly specific test for CSF. Beta-trace protein is another useful marker and may be more rapidly available in some settings [8,17]. A negative test does not exclude a leak if fluid collection was contaminated, insufficient, or obtained during an inactive period. In patients with intermittent leaks, repeated collection or imaging localization may still be necessary [39].

**Table 1:** Practical preoperative checklist.

Domain	Key Elements
Leak confirmation	Beta-2 transferrin or beta-trace protein when fluid is available
Imaging	High-resolution CT; MRI with T2/CISS/FIESTA and FLAIR; consider MRV/CTV
IIH phenotype	Empty sella, optic nerve sheath dilation, globe flattening, Meckel’s cave enlargement, arachnoid pits, multiple defects
Ophthalmology	Papilledema, visual fields, sixth nerve palsy; normal exam does not exclude IIH
OSA	Known diagnosis, CPAP/BiPAP settings, STOP-BANG, polysomnography when feasible
Surgical planning	Defect site, need for Draf IIb/III or transpterygoid approach, vascularized flap, rigid buttress
ICP strategy	Acetazolamide plan, lumbar drain role, neurosurgery involvement, shunt criteria, VSS evaluation

### 6.2. Imaging

High-resolution CT of the skull base and paranasal sinuses is essential for identifying bony defects, skull base thinning, arachnoid pits, lateral sphenoid recess pneumatization, frontal sinus posterior table defects, and multiple areas of erosion [8,17]. MRI complements CT by defining encephaloceles, meningoencephaloceles, soft tissue herniation, and CSF signal. Heavily T2-weighted sequences such as CISS or FIESTA can be particularly useful for localization [8]. FLAIR imaging may help distinguish CSF from secretions. MR venography or CT venography should be considered when venous sinus stenosis, venous hypertension, or IIH is suspected [21,40,41].

Imaging should not focus only on the obvious leak site. Patients with obesity-associated IIH frequently have multiple areas of skull base attenuation [42]. The contralateral anterior skull base, lateral sphenoid recess, posterior table of the frontal sinus, and temporal bone should be reviewed carefully. Missing a second defect may lead to persistent leak, early recurrence, or confusion regarding the adequacy of the repair.

### 6.3. Ophthalmologic and ICP Evaluation

Neuro-ophthalmologic evaluation is valuable, particularly to document papilledema, visual field loss, or sixth nerve palsy [8]. However, a normal eye examination does not exclude elevated ICP in an active CSF leak patient [13]. Direct ICP measurement by lumbar puncture, lumbar drain, or ventriculostomy can help guide therapy but must be interpreted in context [12,25,27]. Opening pressure may be underestimated when an active leak is present, and pressure typically rises after closure [12,13,15]. Postoperative pressure monitoring is a useful method to determine eligibility for pressure reducing measures, such as acetazolamide [26,43,44].

**Figure 4:**
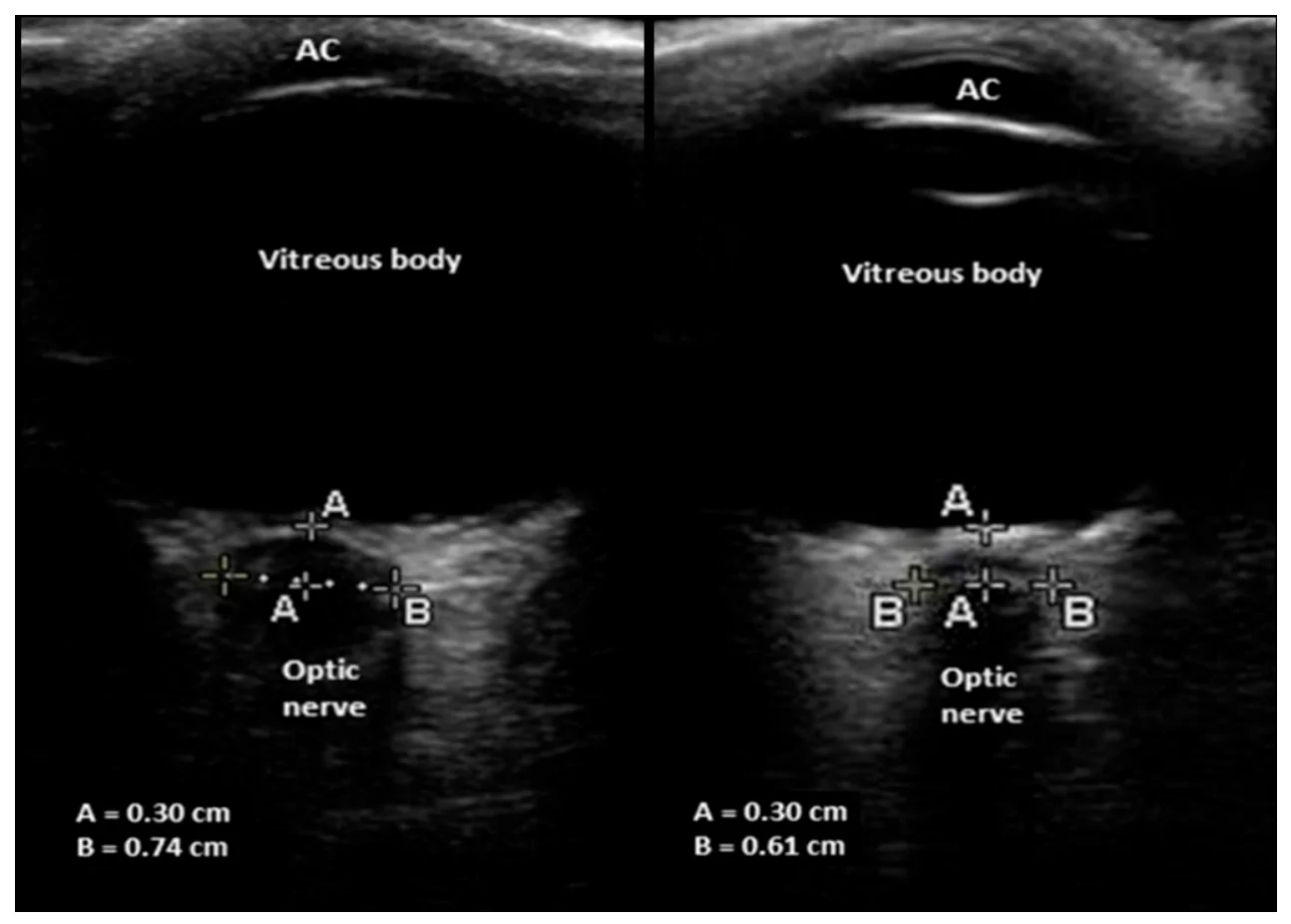
Optic nerve ultrasound revealing a dilated optic nerve sheath of 0.74 cm in a spontaneous CSF leak patient the day after closure of the defect (left). Six hours after administration of 500 mg of acetazolamide, the patient has an optic nerve sheath diameter of 0.61 cm and a drop in CSF pressure of 14 cm H20 according to lumbar drain measurements (right).

Optic nerve sheath diameter (ONSD) ultrasound is a promising noninvasive adjunct. In spontaneous CSF leak patients, ONSD correlates with lumbar drain ICP measurements and may be useful for monitoring response to acetazolamide (Figure 4) [15]. This technique is not yet a replacement for direct pressure assessment in all patients, but it offers an attractive strategy for follow-up and for reducing dependence on invasive monitoring.

### 6.4. OSA Evaluation

OSA should be actively considered in obese skull base patients, especially those with spontaneous CSF leaks or suspected IIH [32]. A documented history of CPAP use should be obtained, but the absence of a prior diagnosis does not exclude clinically significant sleep apnea. Screening tools such as STOP-BANG may identify high-risk patients, and formal polysomnography should be considered when time allows and when results would alter perioperative management [4]. In urgent leak repair, formal sleep testing may not be feasible before surgery, but high-risk patients can still be managed with enhanced monitoring and postoperative airway precautions [19].

## 7. Principles of Management

Management of obesity-associated spontaneous CSF leaks requires two parallel goals: closure of the skull base defect and control of the pressure disorder that caused the defect. Either goal alone is incomplete.

### 7.1. Surgical Closure

Observation, acetazolamide, weight loss, or CSF diversion may reduce pressure, but they do not reliably eliminate the risk of meningitis while an open communication persists between the sinonasal tract and intracranial compartment [18,45]. Endoscopic repair is therefore the standard treatment for most anterior skull base and sphenoid sinus spontaneous CSF leaks [22,46]. Modern endoscopic approaches provide excellent exposure, avoid craniotomy morbidity, and allow site-specific reconstruction [47,48,49].

The surgical approach should be dictated by anatomy. Ethmoid and cribriform defects may be repaired through standard endoscopic sinus approaches. Frontal sinus posterior table defects may require extended frontal sinusotomy, including Draf IIb or Draf III exposure depending on location and access, as well as periorbital suspension for far lateral and supraorbital defects [38,50,51,52]. Lateral sphenoid recess defects often require a transpterygoid approach to obtain adequate exposure lateral to V2 [11,28]. In each setting, the reconstruction should be durable enough to withstand postoperative pressure elevation.

### 7.2. Reconstruction Strategy

**Figure 5:**
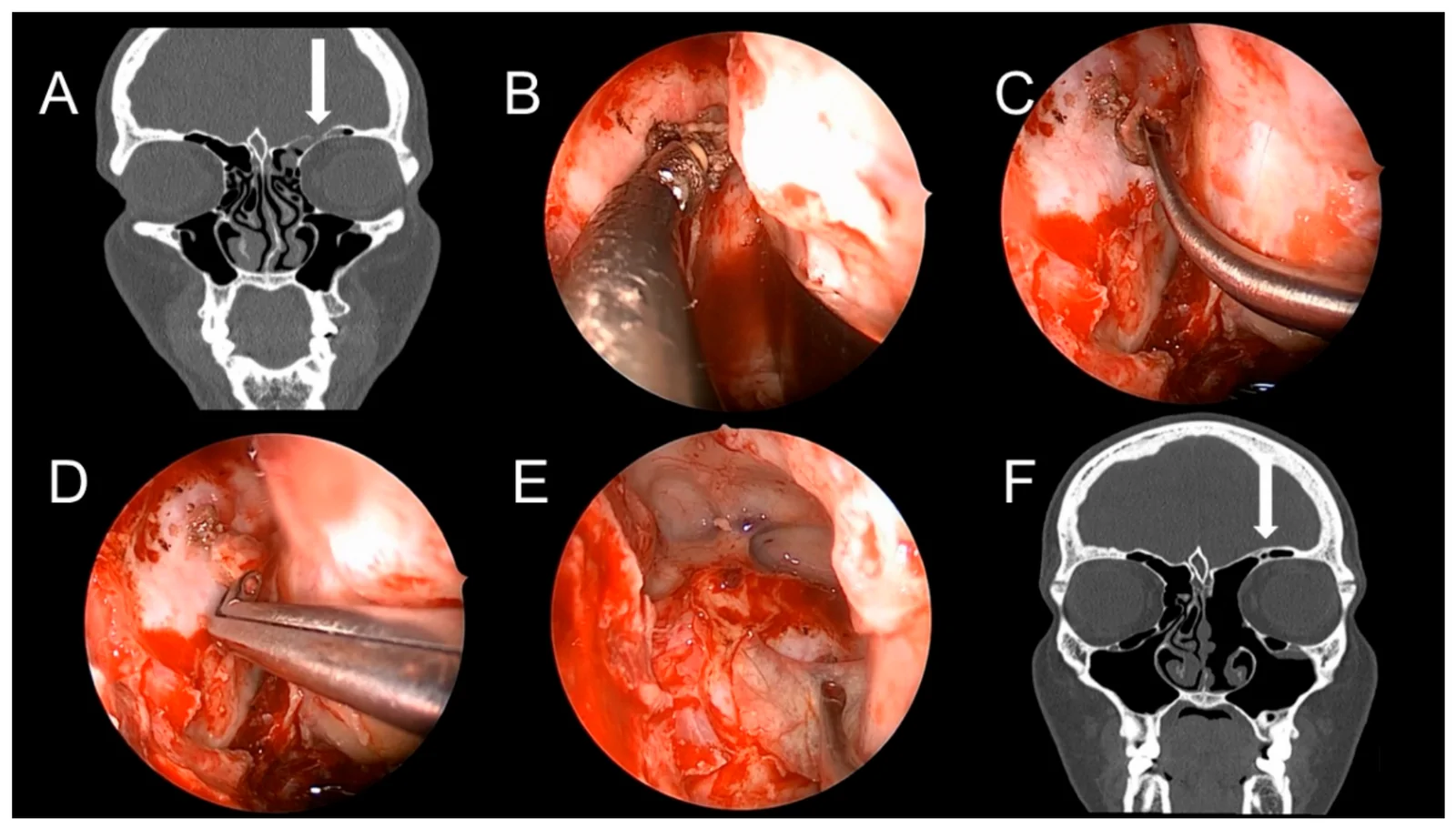
Coronal CT scan of a patient with a defect and encephalocele in a narrow lateral supraorbital recess (**A**). Transnasal endoscopic view after Draf 2B, periorbital suspension with retraction of the orbit with a malleable retractor, and using a coblator to ablate the encephalocele (**B**). Multilayer repair using an underlay porcine small intestine submucosal graft (Biodesign^®^, Cook Medical, Bloomington, IN, USA) placed in epidural fashion (**C**), followed by a bone graft (**D**), and an overlay layer of Biodesign (**E**). One year postoperative CT scan demonstrates complete integration and bone reconstruction of the skull base defect (**F**).

Reconstruction should be tailored to the size, location, flow, and pressure environment of the defect. Small low-flow defects may be successfully treated with multilayer free grafts. Vascularized tissue can be considered for larger defects, high-flow leaks, wide encephaloceles, revision cases, and patients with strong radiographic or measured evidence of IIH [53,54,55,56]. The nasoseptal flap remains a workhorse for many anterior and central skull base defects [57]. Rigid buttress materials, fascia lata, fat, collagen matrix, mucosal grafts, and tissue sealants may be used depending on the defect. The authors prefer to insert bone grafts in the epidural space as a multi-layer reconstruction when possible as this promotes osteoneogenesis and regeneration of the skull base (Figure 5) [58].

The obesity/IIH patient should be considered a high-pressure reconstruction even when the intraoperative leak appears modest. A repair that would be adequate for a small iatrogenic defect may be less durable in a patient with persistent postoperative ICP elevation, OSA-related pressure spikes, chronic cough, or early return to positive airway pressure [4].

### 7.3. Role of Lumbar Drains

The role of lumbar drainage in CSF leak repair remains controversial. Lumbar drains are not required for every repair, and routine drainage solely for diversion has not consistently improved outcomes [59,60,61]. However, in spontaneous CSF leak patients, the value of a lumbar drain is less about diversion and more about diagnosis, localization, intrathecal fluorescein administration, and postoperative ICP monitoring [12,27]. In high-risk patients, pressure data can guide acetazolamide dosing, identify inadequate response to medical therapy, and help determine whether permanent CSF diversion is necessary [12].

## 8. Postoperative ICP Management

The most important management principle is that repair success is linked to long-term ICP control. In a large systematic review and prospective series, primary repair success was higher in cohorts where ICP was evaluated and actively treated with acetazolamide or CSF diversion compared with cohorts without active ICP management [7]. This finding supports an aggressive but rational strategy: repair the skull base and treat the pressure.

### 8.1. Acetazolamide

Acetazolamide is commonly used after spontaneous CSF leak repair because it reduces CSF production. In our prospective study of high-pressure CSF leak patients, oral acetazolamide 500 mg significantly reduced ICP within the 4- to 6-h assessment period [26]. Most patients tolerate the medication, although paresthesias, taste alteration, fatigue, renal considerations, sulfa allergy concerns, electrolyte abnormalities, and pregnancy considerations must be addressed. A basic metabolic panel should be monitored after initiation and with dose escalation.

Acetazolamide should not be viewed as a substitute for repair of an active skull base communication [18]. It is an adjunct to reduce pressure on the repair and lower recurrence risk. Dosing should be individualized based on measured pressure, symptoms, tolerance, renal function, and recurrence risk [26].

Topiramate is an alternative for patients who cannot tolerate acetazolamide or who have comorbid migraine headaches. It inhibits carbonic anhydrase through a mechanism distinct from acetazolamide and promotes weight loss and migraine prophylaxis [1,62]. Preclinical evidence suggests that topiramate and acetazolamide lower ICP via different molecular pathways and may provide additive benefit [63]. However, cognitive adverse effects may limit its use, and its teratogenic potential necessitates careful counseling and avoidance during pregnancy when possible. No prospective studies have compared topiramate with acetazolamide or evaluated its efficacy specifically after skull base CSF leak repair.

### 8.2. Permanent CSF Diversion

Ventriculoperitoneal shunting should be considered in patients with markedly elevated ICP despite acetazolamide, poor response or intolerance to medical therapy, recurrent leaks, multiple defects, extensive skull base attenuation, or severe high-pressure physiology [8,12]. The threshold for shunting should not be based on a single number alone. Patient age, comorbidities, defect burden, radiographic phenotype, occupational strain, OSA severity, and patient preference all matter. In general, patients with very high opening or post-clamp pressures, persistent pressures above the normal range despite acetazolamide, or multiple pressure-related defects deserve early neurosurgical discussion.

### 8.3. Transverse Venous Sinus Stenting

**Figure 6:**
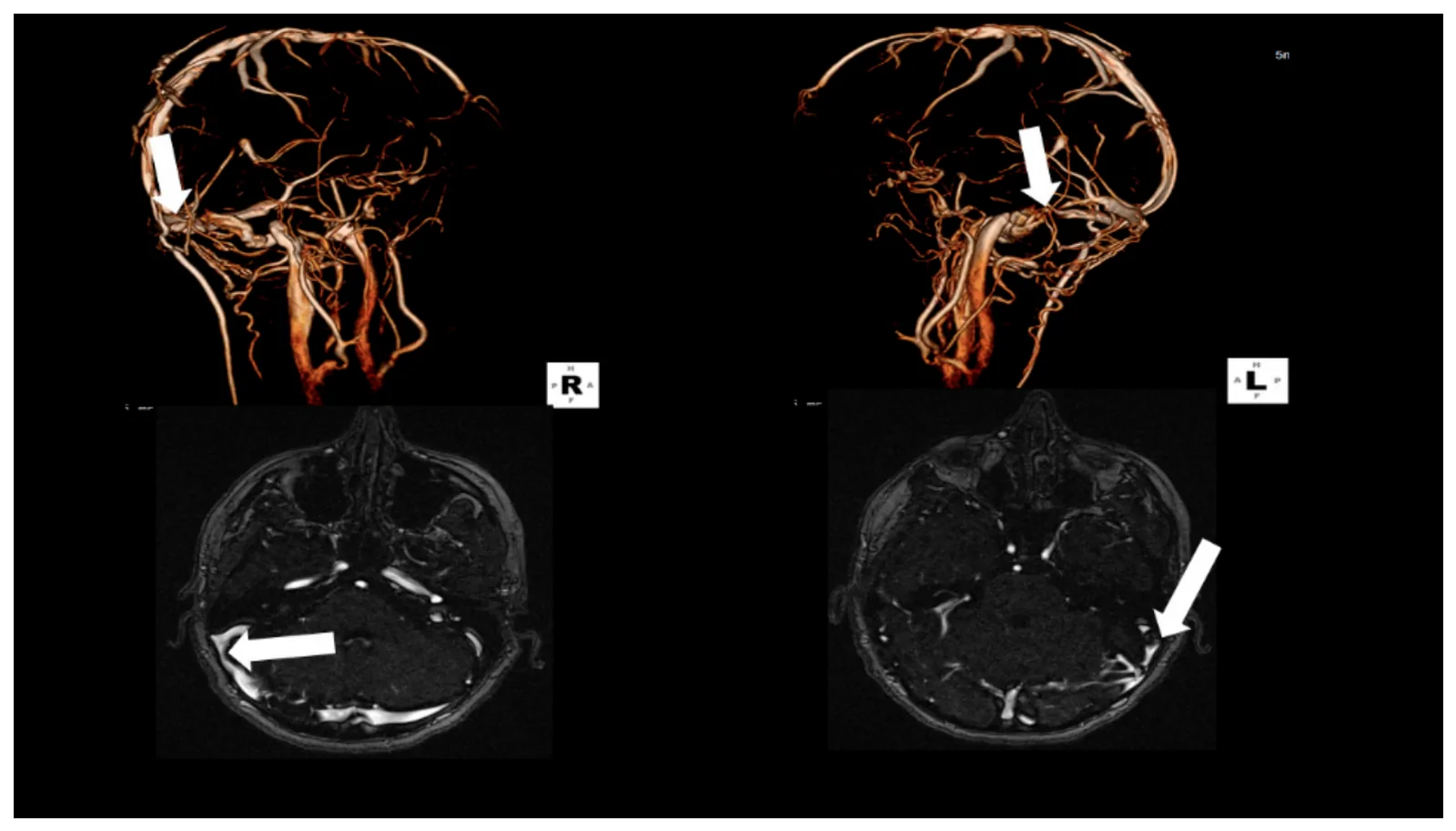
MR venogram demonstrating bilateral transverse sinus stenosis (arrows) in 3-D reconstruction (top panel, R—right view, L—left view) and axial scans (bottom panel).

Transverse-sigmoid venous sinus stenosis is increasingly recognized as part of the IIH phenotype and may be relevant in selected patients with spontaneous skull base CSF leaks [64,65]. In this setting, venous sinus stenting (VSS) offers a pressure-lowering strategy that targets impaired venous outflow rather than CSF production or permanent CSF diversion. The best candidates are patients with radiographic transverse-sigmoid sinus stenosis and a significant trans-stenotic pressure gradient on venous manometry, particularly when there is persistent or recurrent intracranial hypertension after repair, recurrent leak despite acetazolamide, intolerance of medical therapy, multiple pressure-related defects, or a prominent venous phenotype such as pulsatile tinnitus (Figure 6) [21,40]. VSS should not be viewed as a routine replacement for endoscopic closure of an active sinonasal-intracranial communication, because an open CSF fistula still carries risk of meningitis and other intracranial complications [18]. However, it may be a useful adjunct after repair, or in carefully selected patients as part of a multidisciplinary strategy to reduce ICP and avoid or delay shunt placement. Given the current evidence is derived largely from retrospective series and systematic reviews, VSS should be discussed cautiously and should not be presented as a routine component of care for all obese patients with spontaneous CSF leaks [21,40,41]. At present, VSS is best considered a selective adjunct for carefully chosen patients with radiographic transverse-sigmoid sinus stenosis, a significant trans-stenotic pressure gradient on venous manometry, and persistent or recurrent intracranial hypertension despite standard management. It should not replace endoscopic closure of an active sinonasal-intracranial communication, and its role relative to acetazolamide, shunting, weight loss, and skull base repair requires further prospective study. Important limitations include the need for perioperative antiplatelet therapy, uncertainty regarding optimal timing relative to skull base reconstruction, risk of in-stent stenosis or thrombosis, access-site complications, and the limited quality of the current evidence [21,40,41]. Therefore, VSS should be considered in collaboration with neurointerventional radiology, neurosurgery, and otolaryngology, rather than used as a stand-alone algorithmic step for all obese spontaneous CSF leak patients.

### 8.4. Weight Loss and Metabolic Intervention

Weight loss is disease-modifying in IIH and should be incorporated into long-term care. Lifestyle modification, medically supervised weight loss, bariatric surgery, and newer anti-obesity pharmacotherapy may reduce ICP and improve OSA severity [66,67,68]. However, weight loss is not an acute treatment for an open CSF leak and should not delay closure in a patient with ongoing rhinorrhea or intracranial complication risk. The most practical approach is staged: close the leak, control ICP medically or surgically, screen and treat OSA, and then implement durable weight-directed therapy to reduce long-term recurrence risk.

Emerging evidence suggests that GLP-1 receptor agonists may provide additional benefit in IIH beyond weight loss alone. In a small randomized controlled trial, exenatide produced a rapid reduction in ICP that was sustained at 12 weeks, supporting a potential direct effect on CSF dynamics [69]. Subsequent observational and meta-analyses have associated GLP-1 receptor agonist therapy with reduced papilledema, headache burden, visual complications, and procedural intervention [70,71,72]. However, no prospective studies have evaluated whether these agents prevent spontaneous skull base CSF leak recurrence. Until such data are available, GLP-1 receptor agonists should be considered within comprehensive obesity and IIH management rather than as established therapy for CSF leak recurrence prevention.

## 9. OSA and Positive Airway Pressure After Skull Base Surgery

OSA creates one of the most difficult postoperative management issues in skull base surgery. The surgeon is trying to protect the reconstruction from excessive pressure, while the anesthesiologist and sleep physician are trying to protect the patient from hypoxemia, hypercapnia, cardiac strain, and airway obstruction. Both concerns are legitimate.

Known OSA patients can often be managed safely when positive airway pressure (PAP) is withheld temporarily and perioperative monitoring is diligent. However, patients at high risk for undiagnosed OSA may be particularly vulnerable because the surgical team may not implement enhanced monitoring or airway precautions. In one skull base cohort, known OSA was associated with greater need for supplemental oxygen but not increased serious respiratory events or CSF leak, whereas a subgroup at high risk for undiagnosed OSA had increased serious respiratory events and a higher postoperative CSF leak rate [4].

In practical terms, patients with known OSA should have PAP settings, adherence, and disease severity documented before surgery. Patients at high risk for undiagnosed OSA should be identified with screening tools and managed with enhanced postoperative monitoring when formal sleep testing is not feasible before urgent leak repair. PAP resumption should be individualized based on defect size, leak flow, reconstruction type, ICP control, and OSA severity. A patient with severe OSA and major desaturations may require earlier monitored PAP resumption or alternative airway support, whereas a patient with a large high-pressure anterior skull base repair may require a longer period of PAP avoidance. When PAP is restarted, lower pressures, humidification, close follow-up, and patient education regarding recurrent rhinorrhea or pneumocephalus symptoms should be considered.

Practical perioperative strategies include:

- preoperative OSA screening and documentation of CPAP/BiPAP settings [19];
- anesthesia planning with attention to difficult airway risk, opioid-sparing analgesia, and postoperative monitoring [4];
- head-of-bed elevation and avoidance of hypoventilation [19];
- supplemental oxygen, humidification, and continuous pulse oximetry when indicated [4];
- avoidance of early positive pressure when the repair is large, high-flow, tenuous, or under high ICP stress [33];
- consideration of alternative strategies such as mandibular advancement devices, positional therapy, high-flow oxygen, or monitored inpatient observation in selected patients [4,19];
- individualized resumption of CPAP/BiPAP after discussion among otolaryngology, neurosurgery, anesthesia, and sleep medicine [34].

No single restart date is appropriate for all patients. A low flow sellar reconstruction in a patient with severe OSA and significant oxygen desaturation is different from a large anterior skull base or frontal sinus defect repaired under high-pressure conditions in a patient with spontaneous CSF rhinorrhea and multiple defects. Reconstruction type, vascularized flap use, buttress support, leak flow, defect size, ICP control, and OSA severity should determine timing. When PAP is resumed, lower pressures, humidification, close follow-up, and patient education regarding recurrent rhinorrhea or pneumocephalus symptoms should be considered [19,34].

## 10. Obesity and Endoscopic Skull Base Tumor Surgery

Although spontaneous CSF leaks represent the clearest obesity-associated skull base manifestation, obesity and OSA also affect skull base tumor surgery [4]. The concern is that elevated baseline ICP, OSA-related pressure swings, cough, Valsalva, and postoperative PAP may increase pressure on skull base reconstructions [33]. Some studies have identified obesity as a risk factor for postoperative CSF leak after endoscopic endonasal surgery, while other series suggest that modern multilayer reconstruction and vascularized flaps may mitigate this risk [55,56,57,73,74].

The most consistent risk factors for postoperative CSF leak remain intraoperative high-flow leak, large dural defect, expanded approaches, revision surgery, central skull base location, prior radiation, and reconstruction failure [17,75]. Obesity should therefore be considered a patient-specific risk modifier rather than an isolated determinant of outcome. In obese patients undergoing expanded endonasal approaches, the surgeon should have a low threshold for robust multilayer reconstruction, vascularized tissue, rigid buttress when appropriate, and explicit planning for postoperative OSA management.

## 11. Proposed Clinical Algorithm (Table 2)

Step 1: Identify the phenotype. Suspect obesity-associated skull base disease in patients with spontaneous CSF rhinorrhea, encephalocele, lateral sphenoid recess defects, temporal bone CSF leak, multiple skull base defects, empty sella, Meckel’s cave enlargement, optic nerve sheath dilation, or recurrent leak after prior repair.

Step 2: Confirm and localize the leak. Obtain beta-2 transferrin or beta-trace protein when possible. Use high-resolution CT and MRI with T2-weighted sequences. Review the entire skull base, not just the obvious leak site [8].

Step 3: Assess the pressure disorder. Evaluate for IIH symptoms, but do not rely on symptoms or papilledema alone. Obtain neuro-ophthalmology assessment when possible. Consider MRV/CTV. Use lumbar puncture, lumbar drain, or ONSD ultrasound selectively based on risk as part of operative or postoperative protocol [12,15].

Step 4: Screen for OSA. Document known OSA, PAP use, settings, adherence, and disease severity. Screen high-risk patients who do not already have a diagnosis. Surgery should not be postponed solely to determine whether a patient has OSA, as a sleep study may delay appropriate intervention. However, if there is a high degree of clinical suspicion, the patient should be evaluated at least postoperatively, since untreated OSA may worsen intracranial hypertension and contribute to long-term risk of recurrent CSF leak. Postoperative monitoring should also be planned in advance [3,20].

Step 5: Repair the defect. Use site-specific endoscopic exposure and a reconstruction strategy appropriate for a high-pressure environment. Use intrathecal fluorescein and lumbar drain monitoring in spontaneous and other selected high-risk patients [12].

Step 6: Treat postoperative ICP. Use acetazolamide when appropriate. Escalate to CSF diversion for recurrent leak, multiple defects, very high pressures, failed medical therapy, or acetazolamide intolerance. Consider VSS in carefully selected patients with transverse-sigmoid sinus stenosis and a significant pressure gradient [64].

**Figure 7:**
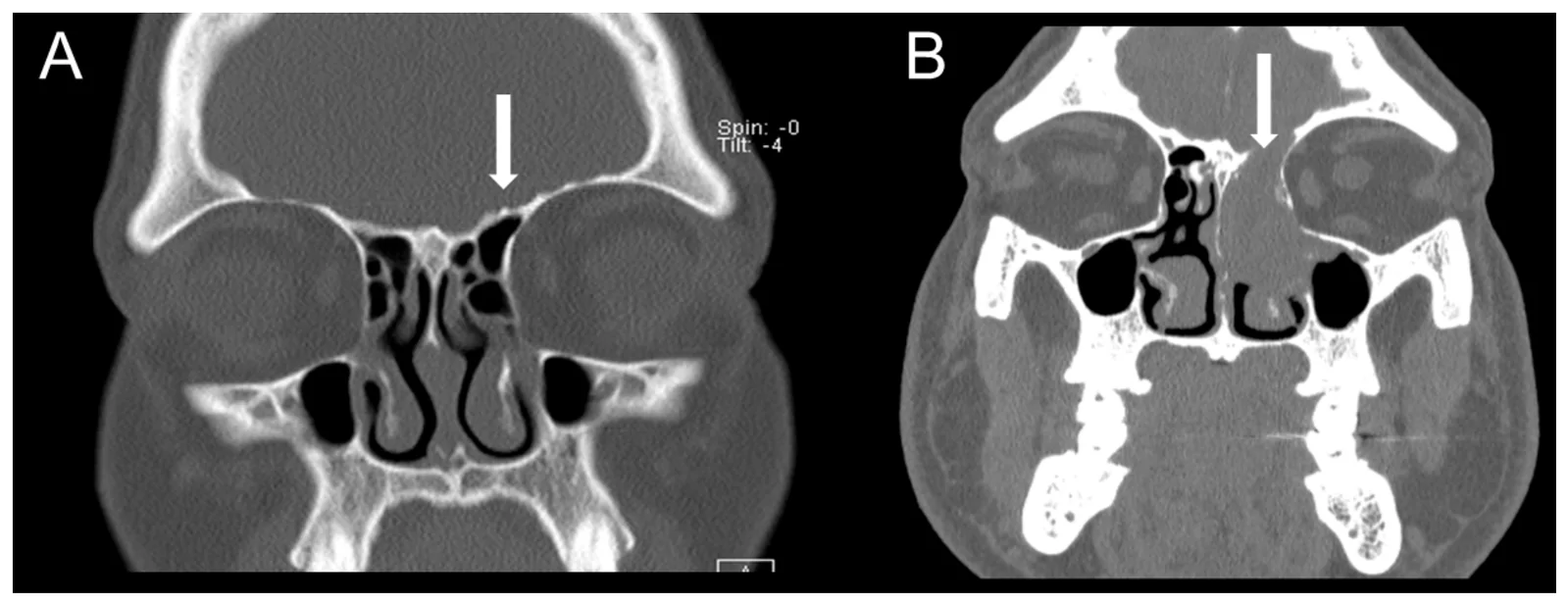
Coronal CT scans from a patient with undiagnosed IIH who underwent endoscopic CSF leak repair of a small right-sided cribriform defect in 2006 (**A**) and later presented in 2022 with a large left frontal sinus posterior table defect and encephalocele (arrow) (**B**), in the setting of untreated IIH.

Step 7: Manage long-term recurrence risk. Address weight loss, OSA treatment, avoidance of severe straining, follow-up imaging and endoscopy, and coordination with neurology, neuro-ophthalmology, neurosurgery, sleep medicine, and obesity medicine. We follow patients yearly with a CT for several years to evaluate their skull base and identify whether they are developing new defects (Figure 7). If pressure is under control long term and there are no skull base changes, we space out the scans for several years.

**Table 2:** Evidence-informed clinical algorithm for obesity-associated skull base disease.

Algorithm Step	Recommendation	Rationale	Approximate Supporting Evidence *
**1. Identify the phenotype**	Suspect obesity-associated skull base disease in patients with spontaneous CSF rhinorrhea, encephalocele, lateral sphenoid recess defects, temporal bone CSF leak, multiple skull base defects, empty sella, Meckel’s cave enlargement, optic nerve sheath dilation, or recurrent leak after prior repair.	Early recognition helps avoid misdiagnosis as rhinitis or isolated anatomic failure and prompts evaluation for an underlying pressure disorder.	**Moderate.** Supported primarily by retrospective cohort studies, radiographic series, systematic reviews, and consistent clinical phenotype descriptions.
**2. Confirm and localize the leak**	Confirm CSF when fluid is available using beta-2 transferrin or beta-trace protein. Localize the defect with high-resolution CT and MRI, and review the entire skull base for additional defects.	Accurate localization guides surgical approach and reconstruction. Multifocal attenuation or additional defects may indicate a pressure-mediated process.	**Moderate to high.** Diagnostic testing and imaging approaches are supported by established skull base practice, diagnostic accuracy studies, retrospective series, and expert consensus.
**3. Assess the pressure disorder**	Evaluate for IIH symptoms and radiographic markers, but do not rely on papilledema or symptoms alone. Consider neuro-ophthalmology evaluation, MRV/CTV, lumbar puncture or lumbar drain pressure monitoring, and selective use of ONSD ultrasound.	Active CSF leak may partially decompress the intracranial compartment and mask classic IIH findings. ICP assessment can guide postoperative medical or surgical pressure management.	**Low to moderate.** Supported by IIH literature, retrospective skull base series, pressure-monitoring studies, small prospective studies, and emerging noninvasive ICP-monitoring data.
**4. Screen for OSA**	Document known OSA, PAP use, settings, adherence, and severity. Screen high-risk patients without a diagnosis and plan postoperative monitoring when formal sleep testing is not feasible before urgent repair.	OSA may contribute to intermittent ICP elevation and increases perioperative respiratory risk. PAP management must be balanced against protection of the skull base reconstruction.	**Low to moderate.** Supported by sleep medicine literature, perioperative OSA data, retrospective skull base cohorts, and limited studies specifically examining OSA in spontaneous CSF leak patients.
**5. Repair the defect**	Use site-specific endoscopic exposure and a reconstruction strategy appropriate for a high-pressure environment. Consider intrathecal fluorescein and lumbar drain monitoring in selected spontaneous or high-risk cases.	Definitive closure eliminates the sinonasal-intracranial communication and reduces meningitis risk. High-pressure patients may require more robust reconstruction than low-risk iatrogenic defects.	**Moderate.** Supported by large retrospective series, systematic reviews, skull base reconstruction literature, and broad expert consensus.
**6. Treat postoperative ICP**	Use acetazolamide when appropriate. Escalate to CSF diversion for recurrent leak, multiple defects, markedly elevated ICP, failed medical therapy, or medication intolerance. Consider VSS only in carefully selected patients with venous sinus stenosis and a significant pressure gradient.	Long-term success depends on treating both the skull base defect and the pressure disorder that contributed to its formation.	**Low to moderate.** Acetazolamide and CSF diversion are supported by IIH literature, retrospective CSF leak studies, systematic reviews, and limited prospective pressure data. Evidence for VSS in this population remains limited and largely retrospective.
**7. Manage long-term recurrence risk**	Address obesity, OSA treatment, avoidance of severe straining, follow-up imaging/endoscopy, and coordination among otolaryngology, neurosurgery, neurology, neuro-ophthalmology, sleep medicine, and obesity medicine.	Obesity, untreated OSA, persistent ICP elevation, and multifocal skull base remodeling may contribute to delayed recurrence or new defect formation.	**Low to moderate.** Supported by IIH weight-loss literature, OSA literature, observational skull base studies, and expert consensus. Prospective skull base-specific data remain limited.

* Evidence levels are approximate and reflect the quality of available literature for each recommendation. Because this is a narrative review rather than a formal guideline or systematic review, evidence was not graded using GRADE methodology. Several recommendations are based on retrospective studies, single-institution series, expert consensus, and extrapolation from IIH, sleep medicine, and skull base reconstruction literature rather than high-level randomized data.

## 12. Future Directions

Several questions remain unanswered. First, prospective studies using systematic polysomnography are needed to define the true prevalence and severity of OSA in spontaneous CSF leak patients. Furthermore, simultaneous or serial ICP monitoring could help determine whether OSA independently contributes to pressure spikes and recurrence risk [3]. The timing of CPAP resumption after endoscopic skull base repair should also be studied by defect type, reconstruction method, and OSA severity rather than by a single arbitrary postoperative interval [34]. Noninvasive ICP monitoring tools such as ONSD ultrasound deserve further validation in postoperative spontaneous CSF leak patients. The role and timing of VSS in spontaneous CSF leak patients with venous sinus stenosis should be clarified prospectively, especially relative to acetazolamide, shunting, and surgical repair. Finally, the effect of sustained weight loss, bariatric surgery, GLP-1-based therapy, and OSA treatment on spontaneous CSF leak recurrence should be evaluated prospectively [2].

A major limitation in the current literature is that many studies examine only one component of the disease process. Otolaryngology studies often focus on repair success, neurology studies focus on IIH symptoms and vision, and sleep medicine studies focus on apnea severity. The next generation of studies should combine these domains. The ideal study would include BMI and body composition, formal polysomnography, standardized imaging markers of IIH, neuro-ophthalmologic assessment, direct or noninvasive ICP measurements, reconstruction details, postoperative PAP timing, weight-loss interventions, VSS candidacy, and long-term recurrence outcomes.

## 13. Limitations

This article has several limitations. First, it is a narrative clinical review rather than a systematic review or guideline, and formal risk-of-bias assessment or pooled quantitative analysis was not performed. Second, some of the cited pressure-monitoring and management data derive from single-institution experience, including work from the authors’ group, which may limit generalizability. Third, the available literature is heterogeneous with respect to patient selection, leak site, reconstruction technique, ICP assessment, OSA diagnosis, PAP timing, weight-loss intervention, and recurrence follow-up. Finally, the proposed algorithm is intended as a practical framework for multidisciplinary evaluation and management, not as a rigid protocol or evidence-graded guideline. Prospective multicenter studies are needed to refine patient selection, standardize ICP and OSA assessment, and determine which interventions most effectively reduce recurrence.

## 14. Conclusions

Obesity-associated skull base disease is a pressure-mediated disorder in which spontaneous CSF leaks and encephaloceles often reflect underlying IIH. Because an active leak can mask classic IIH findings, durable treatment requires both definitive skull base reconstruction and management of elevated ICP.

OSA further complicates care by contributing to intermittent ICP elevation and increasing perioperative respiratory risk, while postoperative PAP therapy must be balanced against protection of the repair. Management should therefore be individualized and comprehensive: identify IIH markers, localize and repair the defect with high-pressure reconstruction, manage ICP, screen and treat OSA, and address obesity as a long-term modifier to reduce recurrence and improve outcomes.

## Data Availability

No new data were created or analyzed in this study. Data sharing is not applicable to this article.

## Abbreviations

The following abbreviations are used in this manuscript:

BiPAP	bilevel positive airway pressure
BMI	body mass index
CISS	constructive interference in steady state
CPAP	continuous positive airway pressure
CSF	cerebrospinal fluid
CT	computed tomography
CTV	computed tomography venography
FIESTA	fast imaging employing steady-state acquisition
FLAIR	fluid-attenuated inversion recovery
GLP-1	glucagon-like peptide-1
ICP	intracranial pressure
IIH	idiopathic intracranial hypertension
MRI	magnetic resonance imaging
MRV	magnetic resonance venography
ONSD	optic nerve sheath diameter
OSA	obstructive sleep apnea
PAP	positive airway pressure
STOP-BANG	snoring, tiredness, observed apnea, high blood pressure, body mass index, age, neck circumference, and gender
V2	maxillary division of the trigeminal nerve
VSS	venous sinus stenting
